# Determination of brain death for adult patients with ECMO

**DOI:** 10.1186/cc14646

**Published:** 2015-03-16

**Authors:** I Ceylan, R Iscimen, E Cizmeci, N Kelebek Girgin, F Kahveci

**Affiliations:** 1 Uludag University Faculty of Medicine, Bursa, Turkey

## Introduction

ECMO support in ARDS is an emerging strategy when conventional treatment modalities fail. ECMO has advantages on oxygenation and circulation but also it has some unfavorable effects. The most serious complication is brain death due to cerebrovascular hemorrhage. An apnea test is the most important component in confirming brain death. For patients supported by ECMO, apnea testing remains challenging. Brain-death diagnosis is often made without an apnea test.

## Methods

We present two cases who receive V-V ECMO support after progression to ARDS. After initiation of ECMO we used sedation to prevent movement and improve adaptation to mechanical ventilation. Also we used anticoagulation with heparin to prevent thromboembolic events and ECMO circuit occlusion. On daily follow-up we noticed that patients had lost their pupil reactions to light. Their sedation was ceased and a computed brain tomography was performed. Both patients had intracerebral hemorrhage. We decided to determine brain death with apnea tests. We increased ECMO blood flow and fiO_2_ and then decreased sweep gas flow and disconnected the patient from mechanical ventilation respectively. In one patient we did not see any spontaneous breathing efforts after carbon dioxide retention. We concluded that the apnea test was successful and confirmed brain death. On the other hand, we confirmed the brain death of the other patient with cerebral angiography due to the occurrence of hypoxia and hypotension during apnea testing.

## Results

We experienced some challenges while determining brain death in patients under ECMO support for ARDS. It is challenging to conduct the apnea testing during ECMO support. Auxiliary tests are required for patients who cannot tolerate the changes needed to conduct the apnea test. With increasing use of ECMO therapies, clinicians may come face to face with more complicated life-ending decisions.

## Conclusion

Current guidelines do not include brain death criteria using supportive therapies such as ECMO and therefore should be updated.
